# Supplementary material for: Anticancer and Targeting Activity of Phytopharmaceutical Structural Analogs of a Natural Peptide from Trichoderma longibrachiatum and Related Peptide-Decorated Gold Nanoparticles
Source: Int J Mol Sci. 2023 Mar 14;24(6):5537. doi: 10.3390/ijms24065537 (PMC10057332; doi:10.3390/ijms24065537)
Supplement: Supplementary file 1 [file ijms-24-05537-s001.zip › ijms-2254638-supplementary.pdf]

Supporting Information

Anticancer Activity of Structural Analogs of a Phytochemical Peptide from *Trichoderma longibrachiatum* and Related Peptide-Decorated Gold Nanoparticles

Francesca Moret[1], Luca Menilli[1,#], Celeste Milani[1,#], Giorgia Di Cintio[2], Chiara Dalla Torre[2], Vincenzo Amendola[2], Marta De Zotti[2,*]

[1] Department of Biology, University of Padova, 35131 Padova, Italy; francesca.moret@unipd.it; luca.menilli@unipd.it; celeste.milani@studenti.unipd.it

[2] Department of Chemical Sciences, University of Padova, 35131 Padova, Italy; giorgia.dicintio@studenti.unipd.it; chiara.dallatorre.2@studenti.unipd.it; vincenzo.amendola@unipd.it; marta.dezotti@unipd.it

* Correspondence to Prof. Marta De Zotti: marta.dezotti@unipd.it
\# The authors contributed equally

TABLE OF CONTENTS

1. **Table S1: plant protection properties of trichogin analogs**
2. **HPLC profiles and HR-ESIMS spectra for the new peptide sequences synthesized in the present work**
3. **GNP characterizations**
4. **CD spectra of the new peptide sequences**
5. ***In vitro* analysis**

1. Table S1: plant protection properties of trichogin analogs

Acronym	PPP
**TRIC**[b]	poor activity against plant pathogens [22]
**Api8-NH2**	new sequence. [21][b]
**Leu4-NH2**	not yet studied (new sequence)
**K2569-Lol**	active against Gram- bacterial plant pathogens [23]
**K259-NH2**	active against Gram- bacterial plant pathogens [23]
**K259-Lol**	active against *Pyricularia orizae* [22]
**K25-Lol**	active against *B. cinerea* [21]
**K56-Lol**	active against *B. cinerea* and *Plasmopara viticola* [21,25]
**K6-NH2**	active against *B. cinerea* and *P. orizae* [22,24]
**K2-NH2**	active against *P. orizae* [22]
**Api8-FITC**	not yet studied (new sequence)
**Leu4-FITC**	not yet studied (new sequence)
**Api8-SH**	not yet studied (new sequence)
**Leu4-SH**	not yet studied (new sequence)

2. HPLC profiles and ESI-MS spectra of the newly synthesized peptides

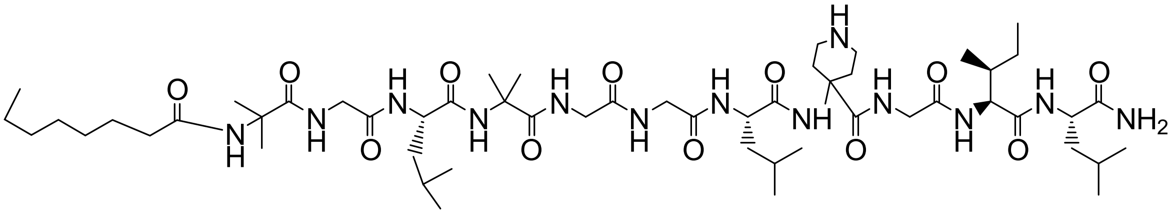

Chemical Formula: $C_{54}H_{97}N_{13}O_{12}$
Exact Mass: 1119.74

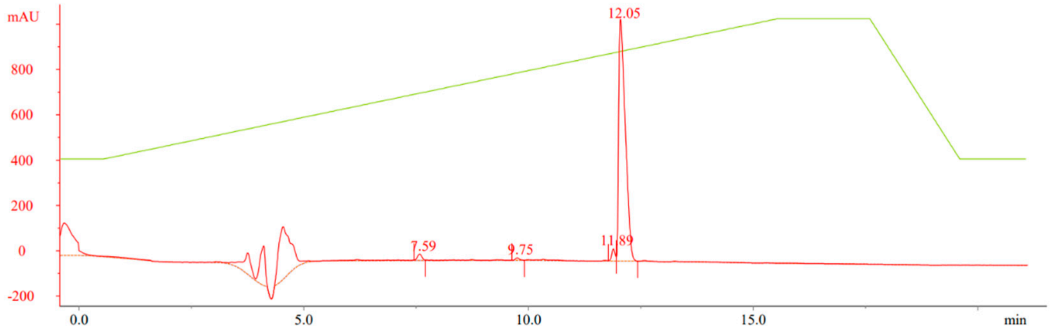

Fig S1. Api8-NH2. Phenomenex C18 column; gradient: 40%-100%B in 15 min, flux 1 mL/min, 216nm. Purity 95%

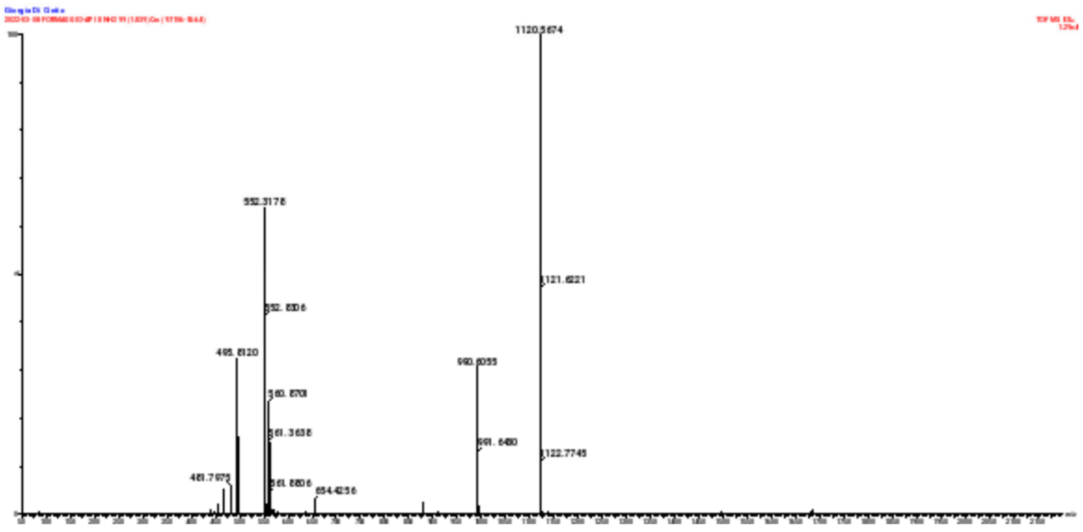

Fig. S2. HR-ESIMS Api8-NH2. $[MW+H]^{+}_{found}$ = 1120.5674 g/mol ; $[MW+2H]^{++}_{found}$ = 560.6701 m/z; $[MW\text{-}Oct+H]^{+}$ = 990.6055 m/z; $[MW\text{-}Oct+2H]^{++}$ = 495.8120 m/z.

Api8-FITC

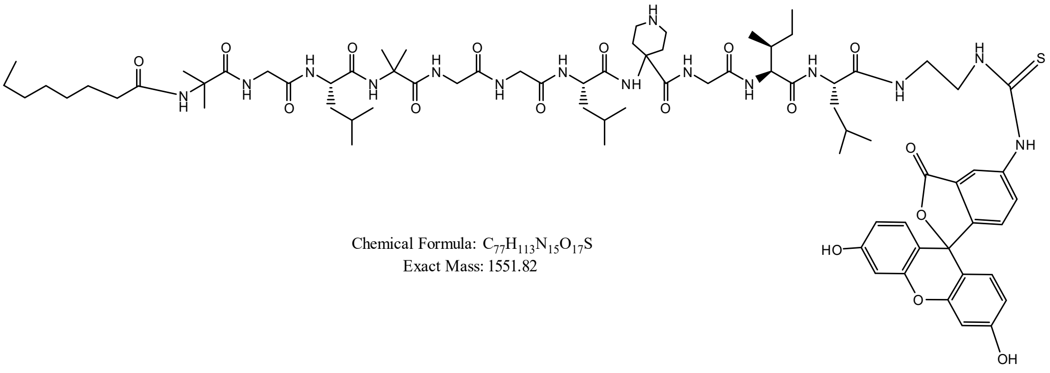

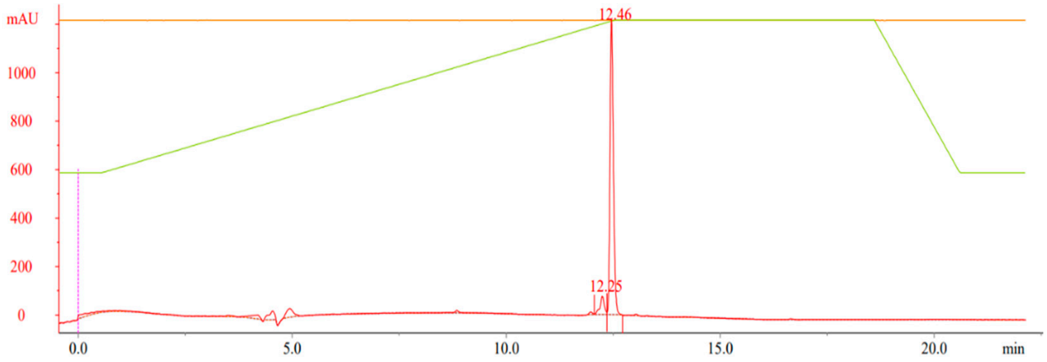

Fig S3. HPLC Chromatogram Api8-FITC. Purity: 95%

ESI-HRMS

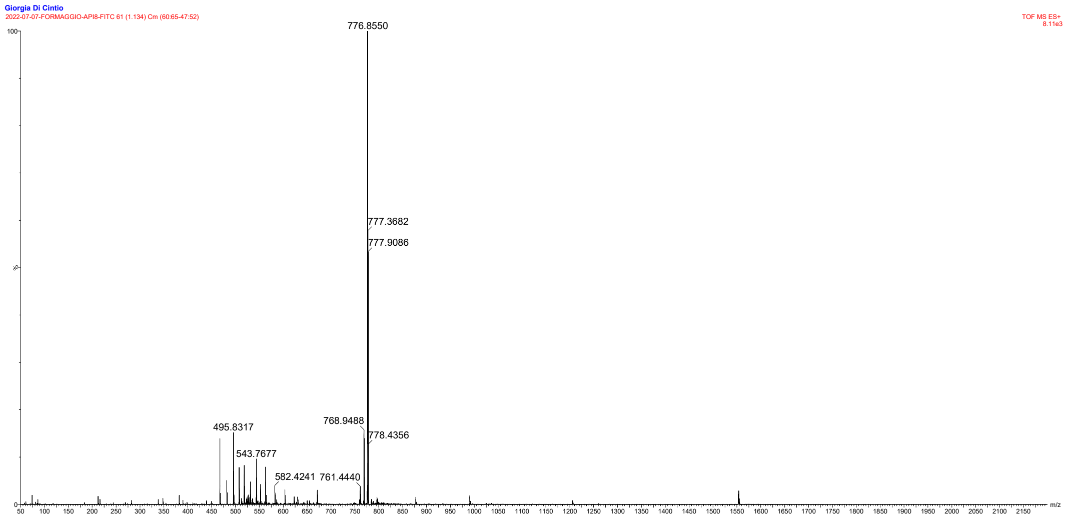

Fig. S4. ESI-HRMS Api8-FITC. $[MW+H]^{+}{}_{found}$ = 1552.8671 m/z; $[MW+2H]^{2+}{}_{found}$ = 776.8550 m/z.

Api8-SH

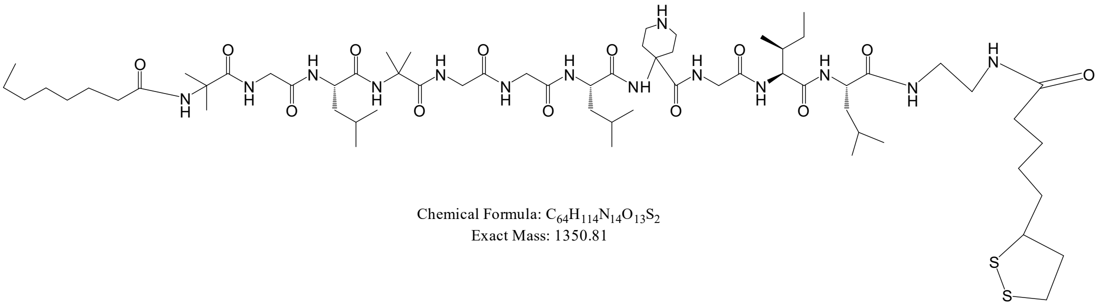

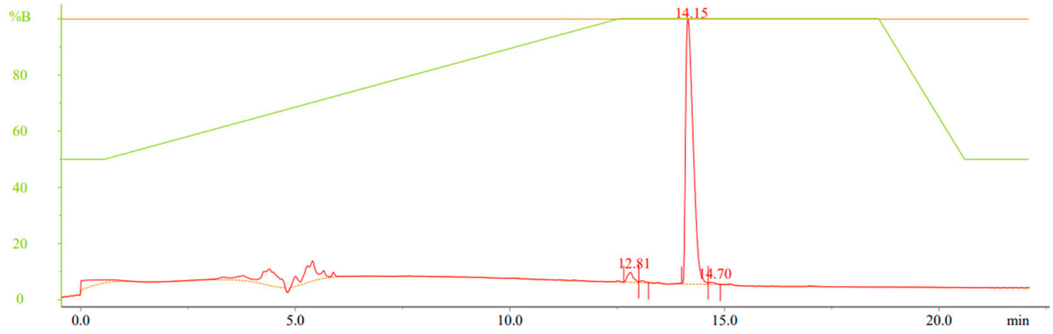

Fig. S5. HPLC chromatogram Api8-SH. Phenomenex C4 40-100%B in 13 min, flux 1 ml/min, wavelength 216 nm. Purity: 96%,

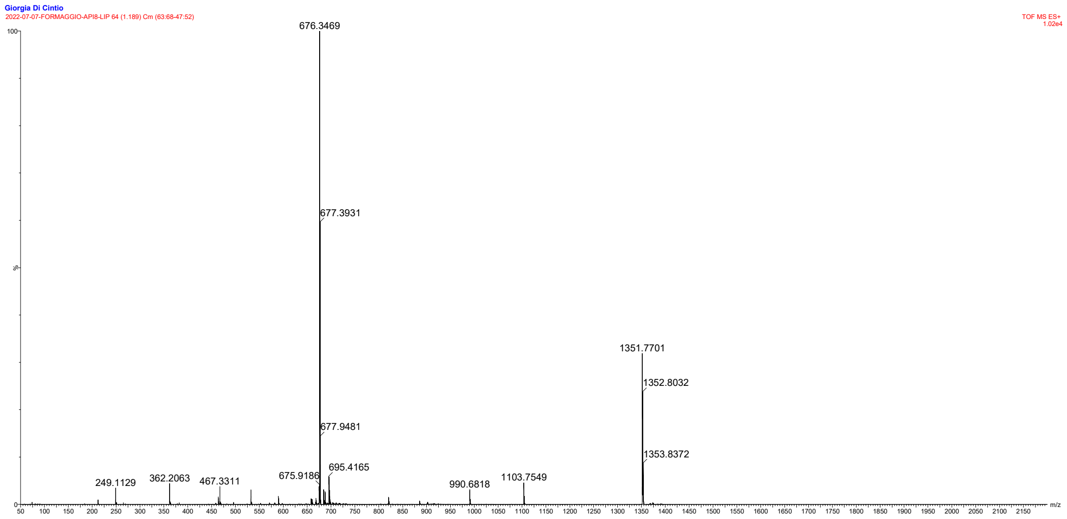

Fig. S6. ESI-HRMS Api8-SH. $[MW+H]^{+}_{found}$ = 1351.7701 m/z ; $[MW+2H]^{++}_{found}$ = 676.3469 m/z

Leu4-NH2

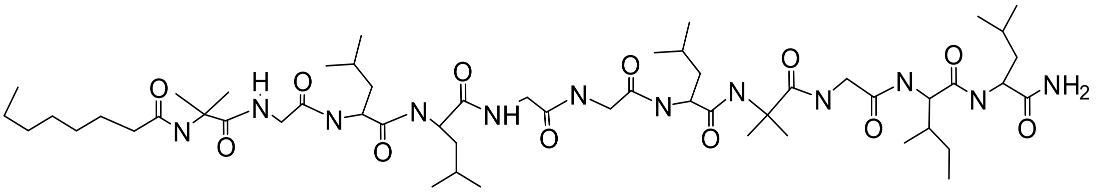

Chemical Formula: $C_{54}H_{98}N_{12}O_{12}$
Exact Mass: 1106.74

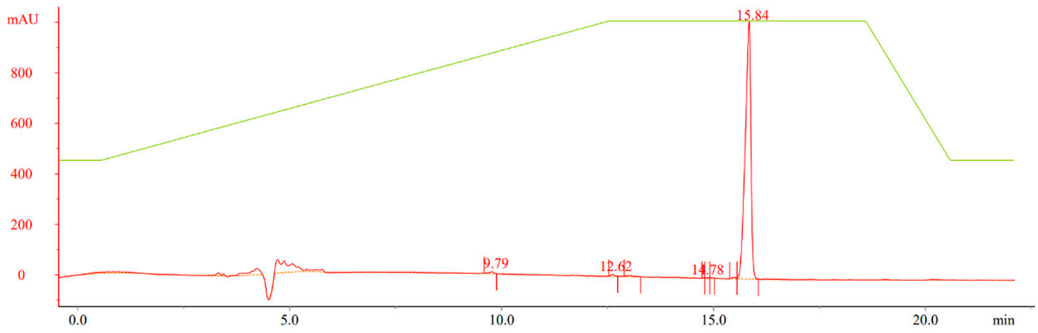

Fig. S7. HPLC Chromatogram Leu4-NH2. Phenomenex C18 50-100%B in 13 min, flux 1 ml/min, wavelength 216nm. Purity 98%.

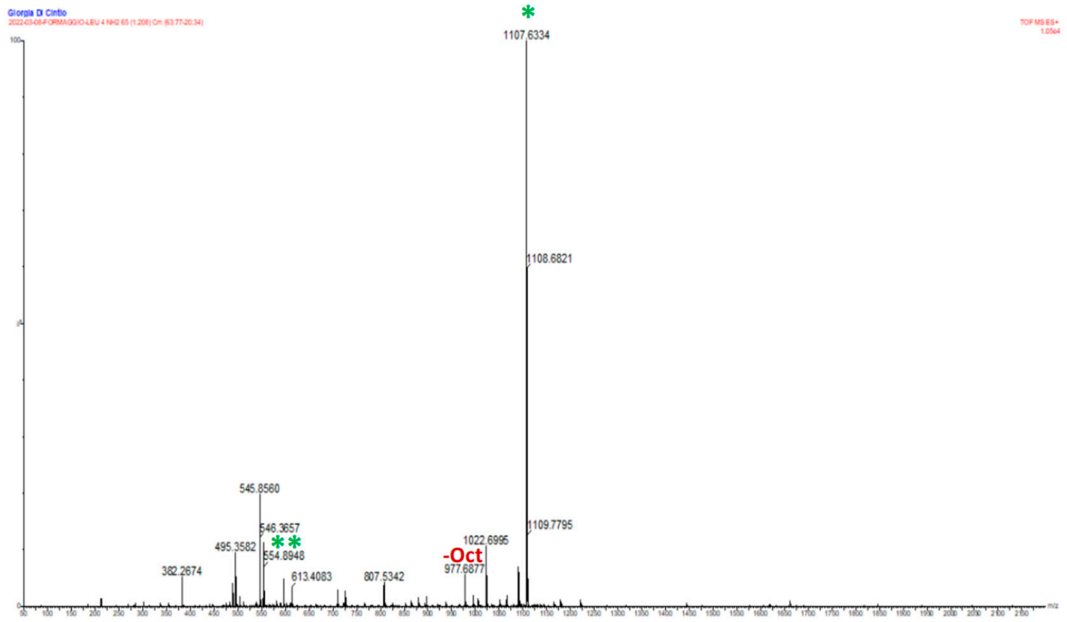

Fig. S8. ESI-HRMS Leu4-NH2. $[MW+H]^{+}_{found}$ = 1107.6334 m/z; $[MW+2H]^{++}_{found}$ = 554.8948 m/z; $[MW\text{-}Oct+H]^{+}_{found}$ = 977.6877 m/z.

Leu4-FITC

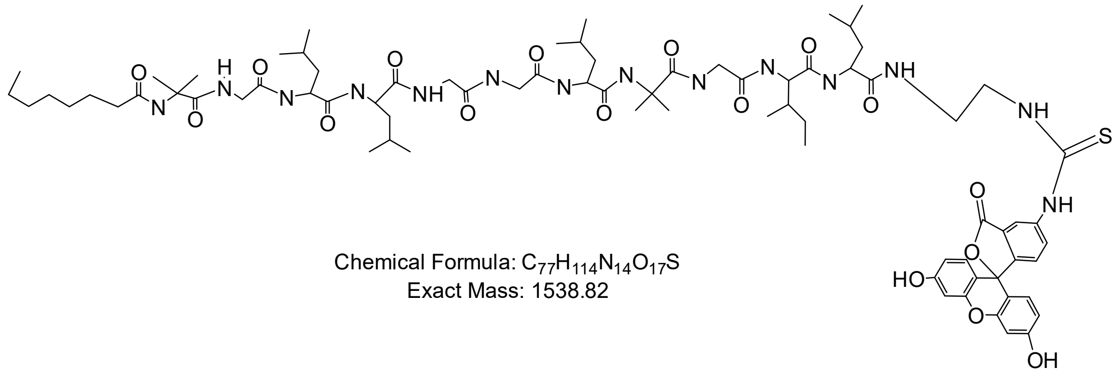

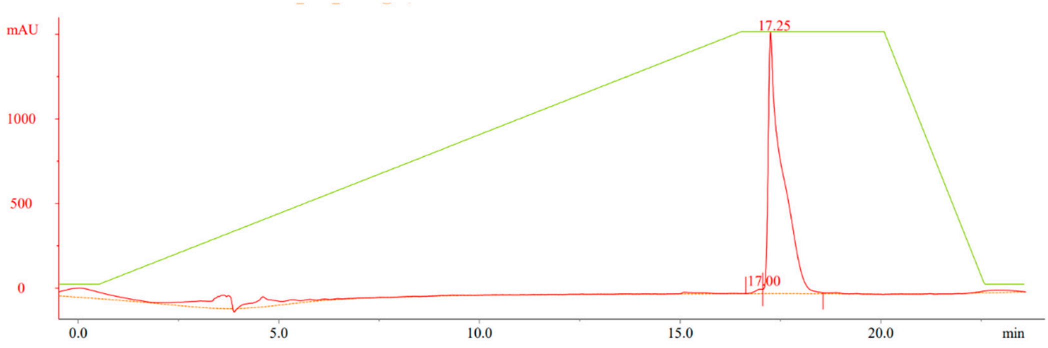

Fig S9. HPLC Chromatogram Leu4-FITC. Phenomenex C18 10-100%B in 15 min, flux 1 ml/min, wavelength 216nm. Purity 98%.

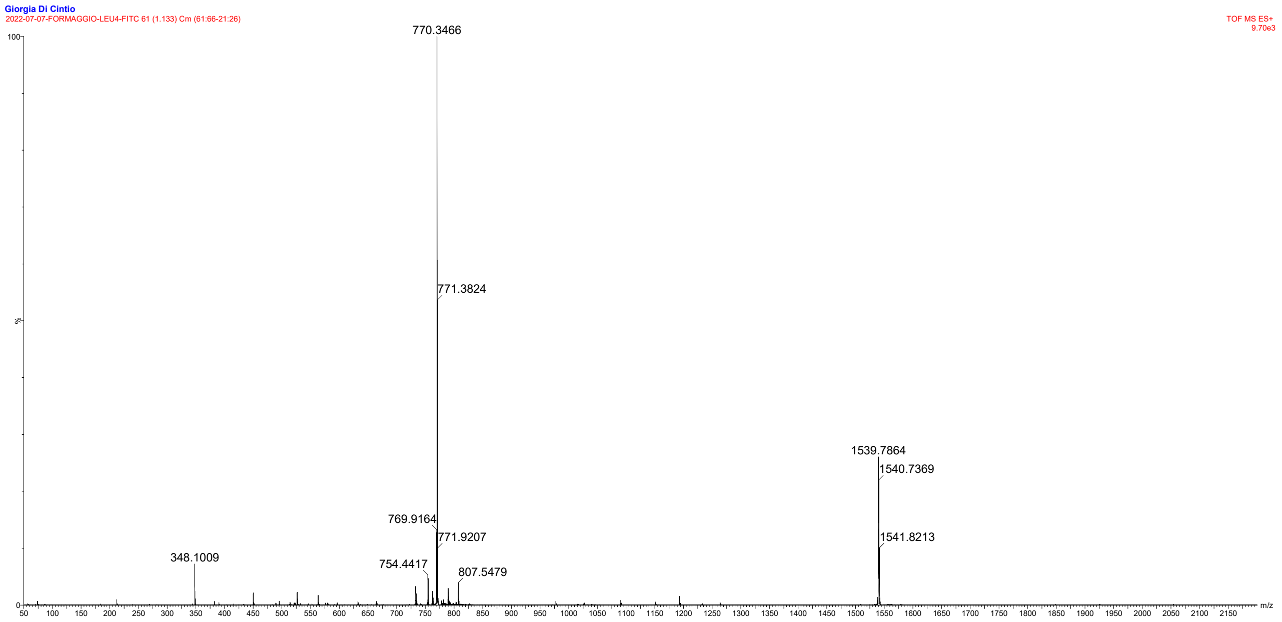

Fig. S10. ESI-HRMS Leu4-FITC. $[MW H]^{+}_{found}$ = 1539.7864 m/z; $[MW+2H]^{++}_{found}$ = 770.3466 m/z

Leu4-SH

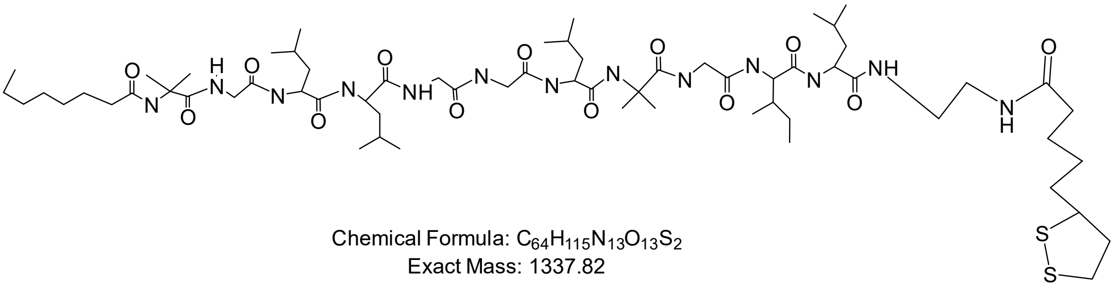

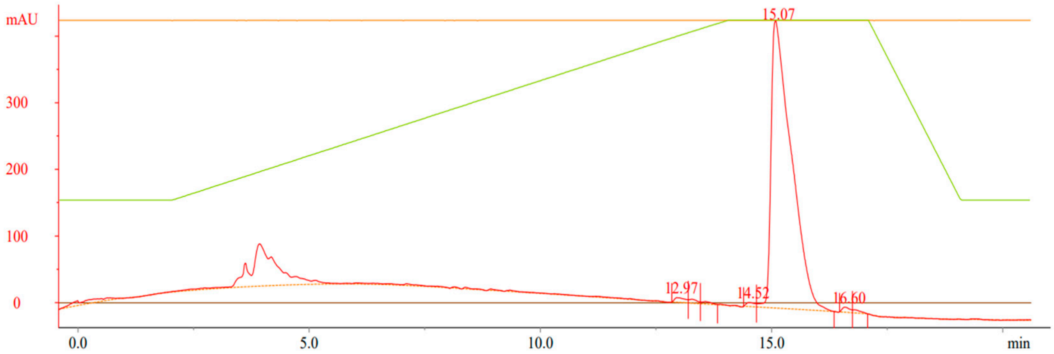

Fig. S11. HPLC chromatogram Leu4-SH. Phenomenex C4 40-100%B in 10 min, flux 1 ml/min, wavelength 216 nm. Purity 96%.

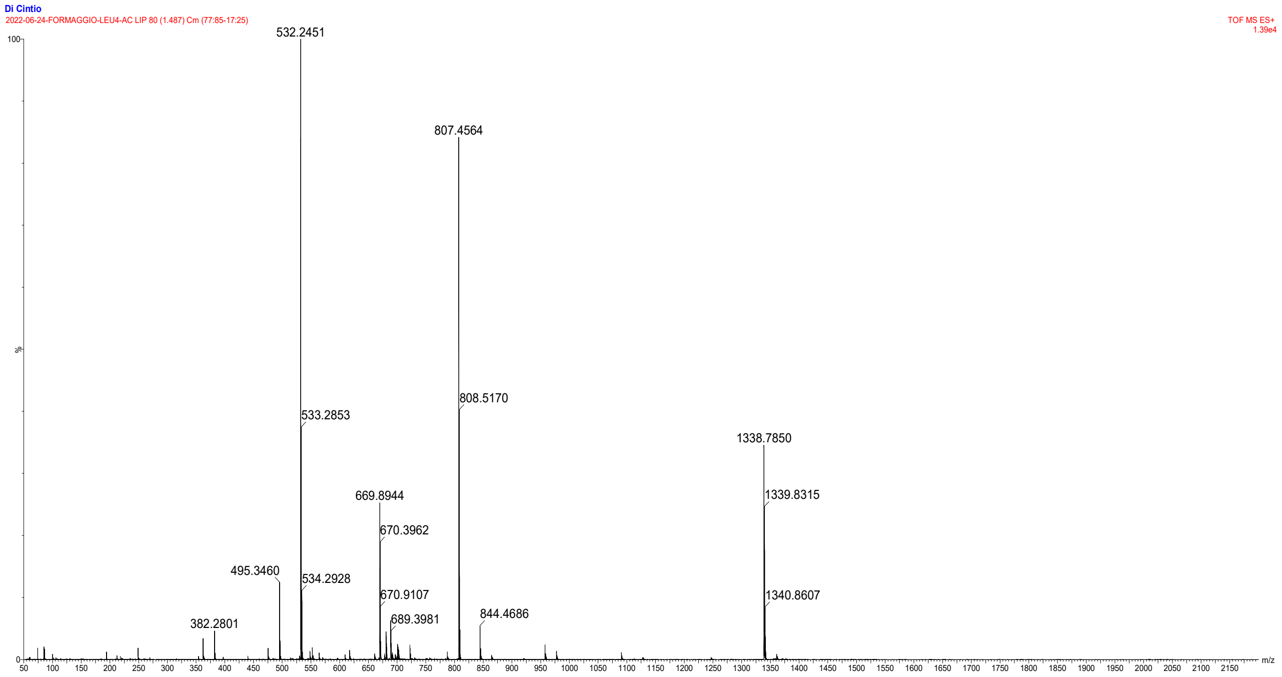

Fig. S12. ESI-HRMS Leu4-SH. $[MW+H]^{+}_{found}$ = 1338.7850 m/z; $[MW+2H]^{++}_{found}$ = 669.8944 m/z; mass fragmentation: 532.25+807.46 = 1339.71

2. GNP characterizations

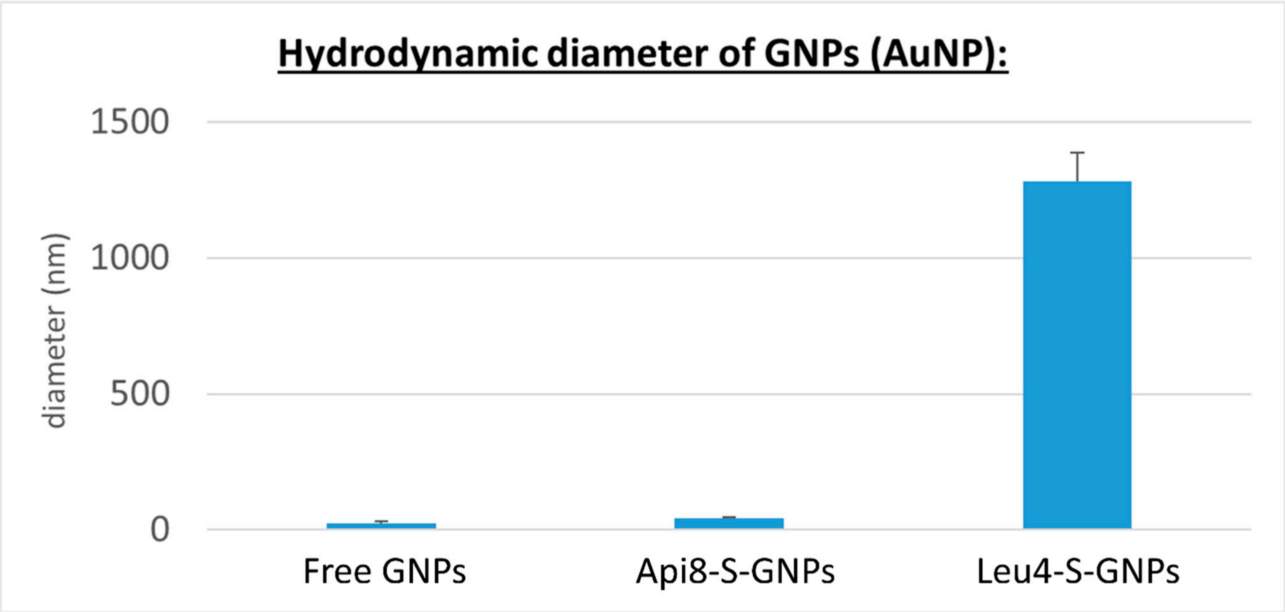

Figure S13. Average diameter of the GNPs obtained by dynamic light scattering.

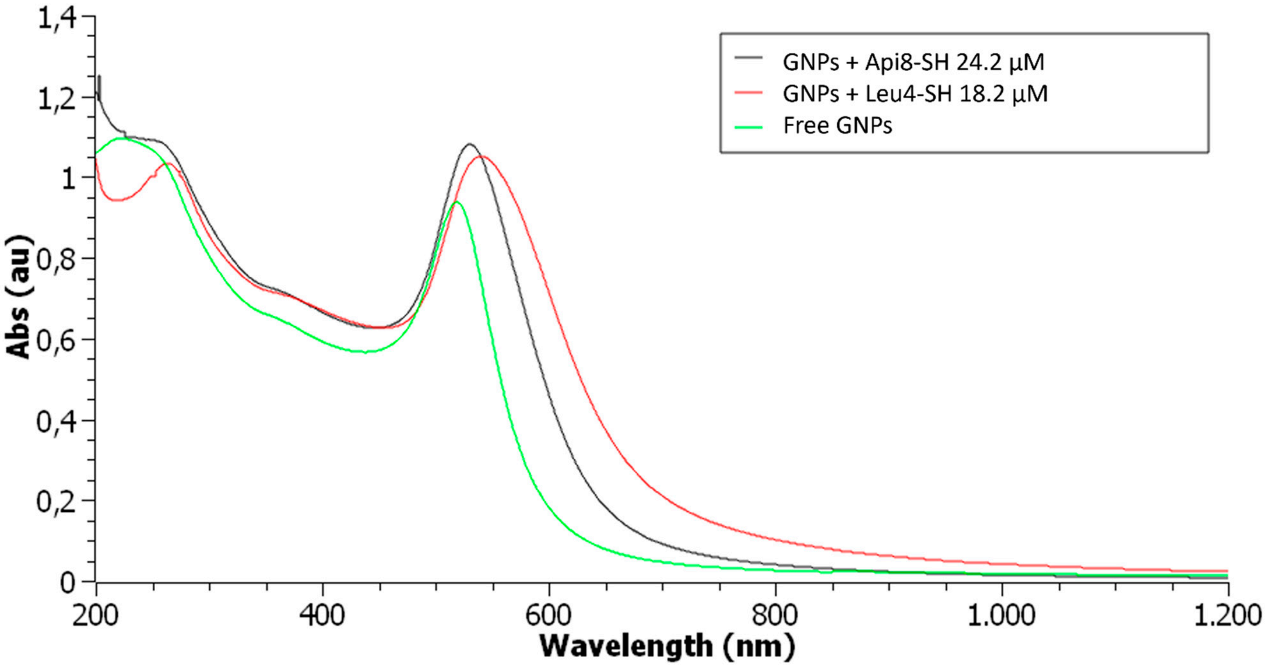

Figure S14. Absorption spectra of GNPs before (green curve) and after five-hour incubation in the presence of Leu4-SH 18.2 µM (red curve) or Api8-SH 24.2 µM (black curve).

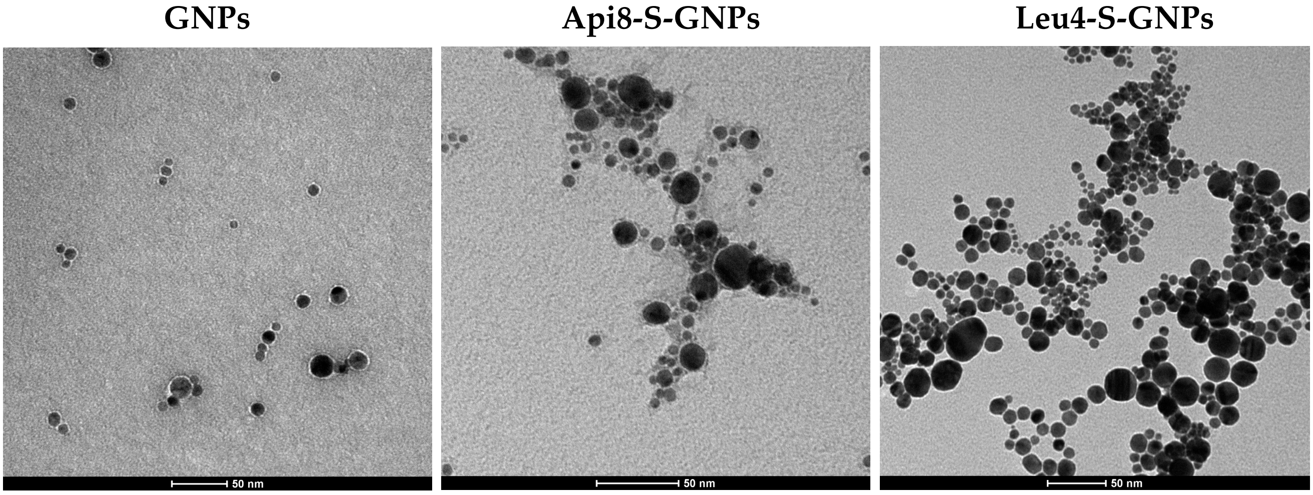

Figure S15. TEM images of GNPs suspensions before (left column) and after incubation with Api8-SH (central column) or Leu4-SH (right column).

3. CD spectra of the new peptide sequences

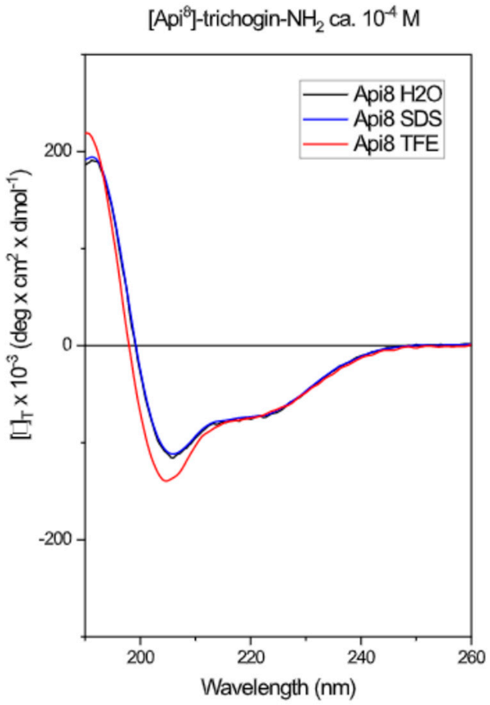

Figure S16. CD Spectra of Api8-NH_2 in sodium dodecyl sulfate (SDS) 100mM (blue), H_2O (black) and 2,2,2-trifluoroethanol (TFE) (red). Peptide concentration 0.1mM.

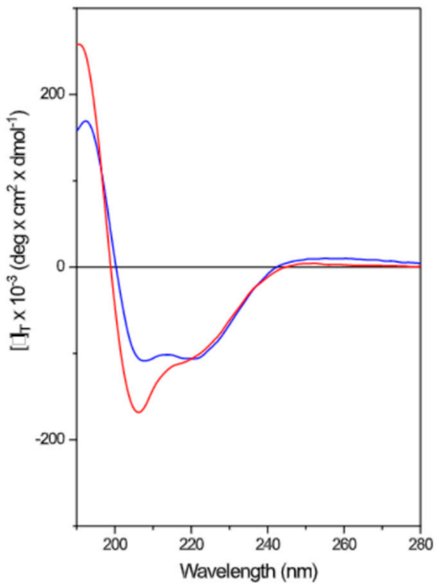

Figure S17. CD Spectra of Api8-FITC in SDS 100mM (blue) and TFE (red). Peptide concentration 0.1mM.

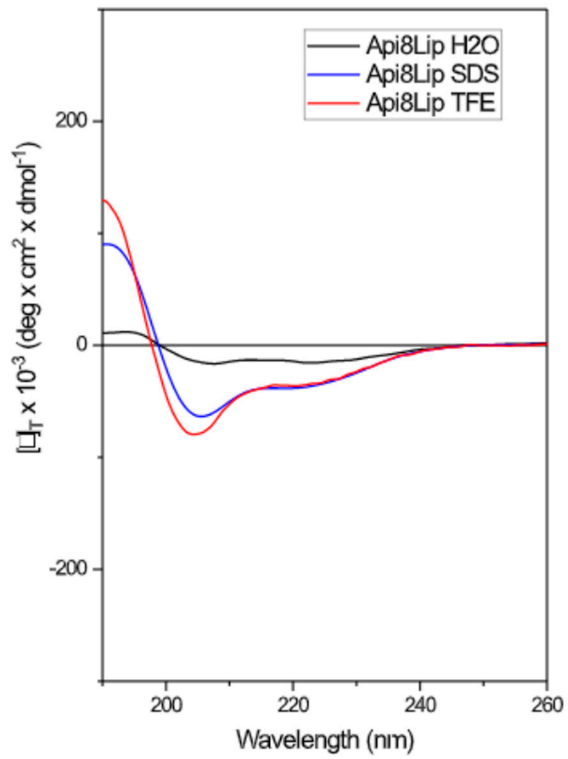

Figure S18. CD Spectra of Api8-SH in SDS 100mM (blue), H_2O (black) and TFE (red). Peptide concentration 0.1mM.

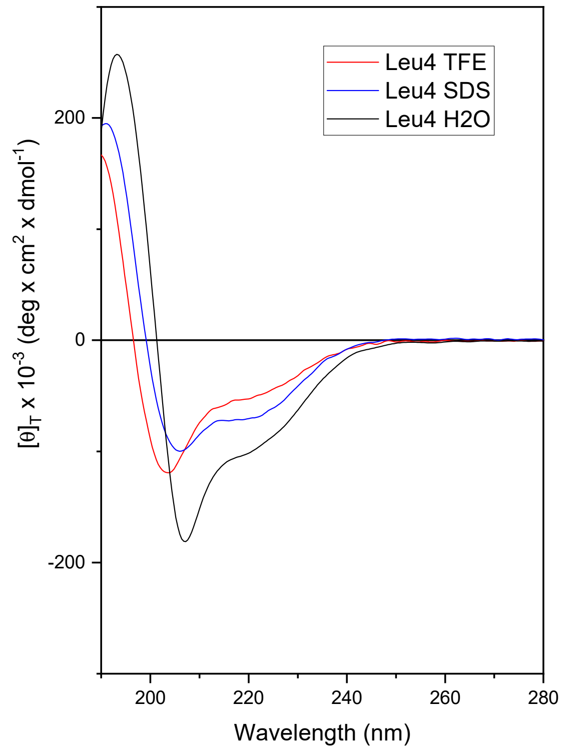

Figure S19. CD Spectra of Leu4-NH2 in SDS 100mM (blue), H_2O:CH_3OH 9:1 (black) and TFE (red). Peptide concentration 0.1mM. Methanol was added to improve solubility

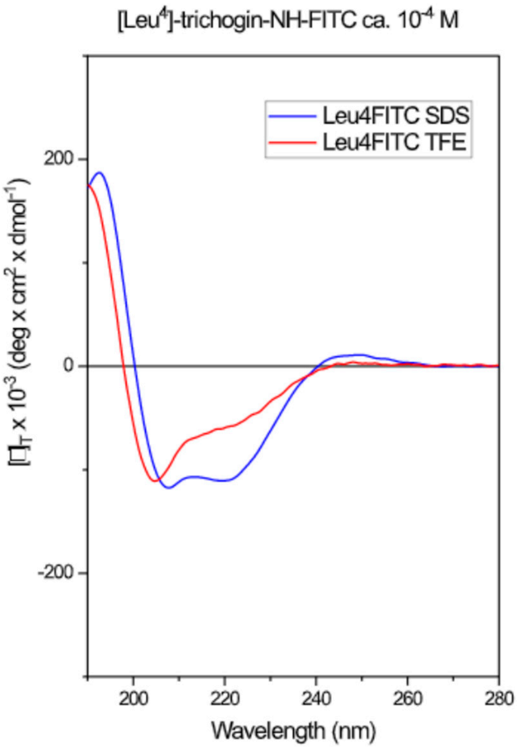

Figure S20. CD Spectra of Leu4-FITC in SDS 100mM (blue) and TFE (red). Peptide concentration 0.1mM.

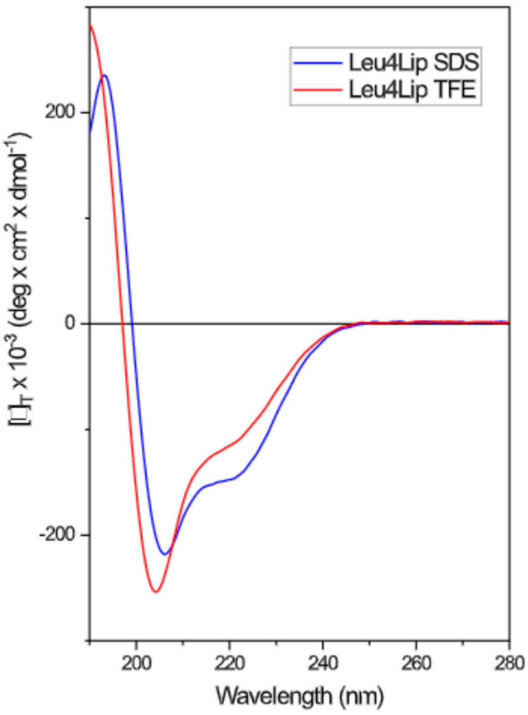

Figure S21. CD Spectra of Leu4-SH in SDS 100mM (blue) and TFE (red). Peptide concentration 0.1mM.

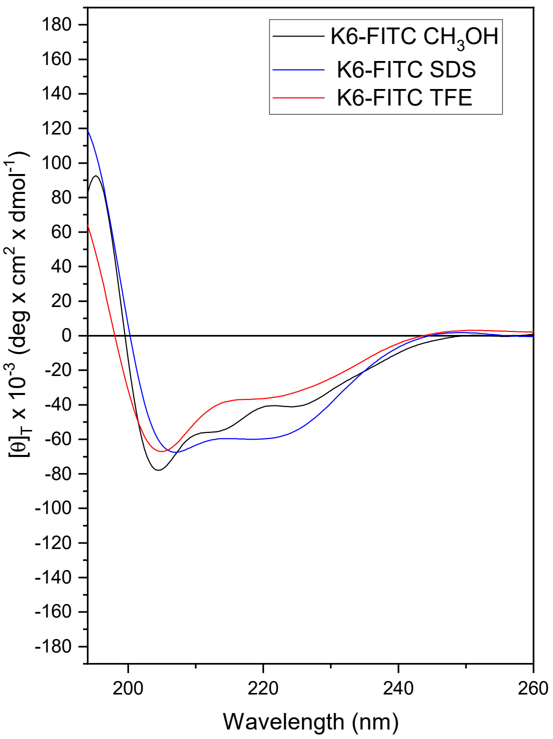

Figure S22. CD Spectra of K6-FITC in SDS 100mM (blue) and TFE (red). Peptide concentration 0.1mM.

4. *In vitro* analysis

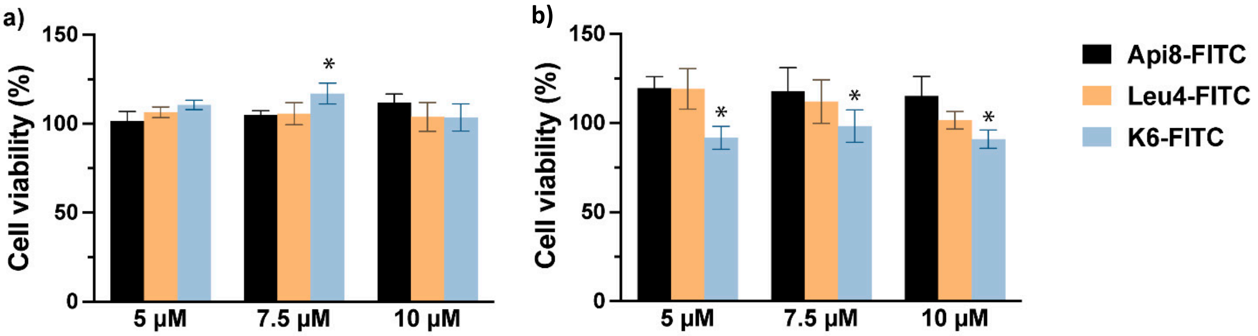

Figure S23. Cytotoxicity of FITC-conjugated peptaibols toward triple negative breast cancer cells MDA-MB-231 (a) and normal breast cells MCF-10A (b). Cells were exposed to increasing concentrations of peptaibols for 24 h and viability was measured with the MTS assay at the end of the incubation time. Data are expressed as mean percentage ± SD of at least two independent experiments, carried out in triplicate. *: $p<0.001$, significantly different from controls (t-test).

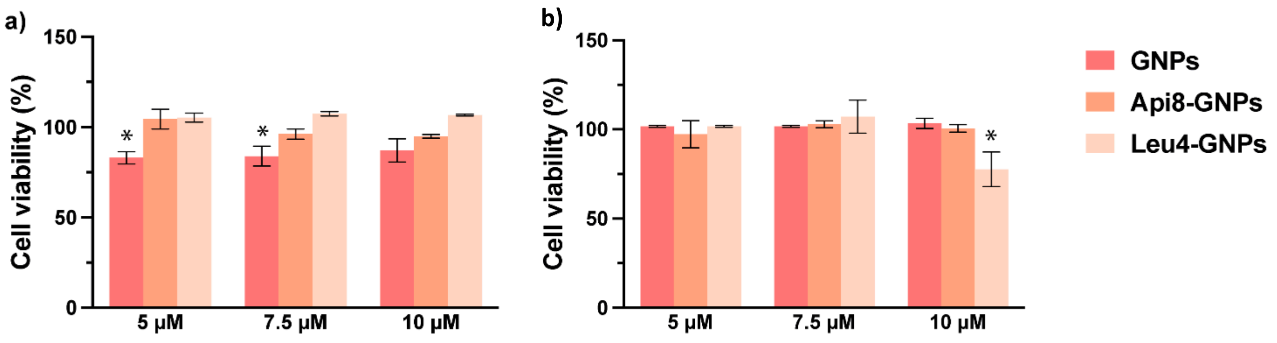

Figure S24. Cytotoxicity of GNP formulations toward triple negative breast cancer cells MDA-MB-231 (a) and normal breast cells MCF-10A (b). Cells were exposed to increasing concentrations of GNPs for 24 h and viability was measured with the MTS assay at the end of the incubation time. Data are expressed as mean percentage ± SD of at least two independent experiments, carried out in triplicate. *: $p<0.001$, significantly different from controls (t-test).

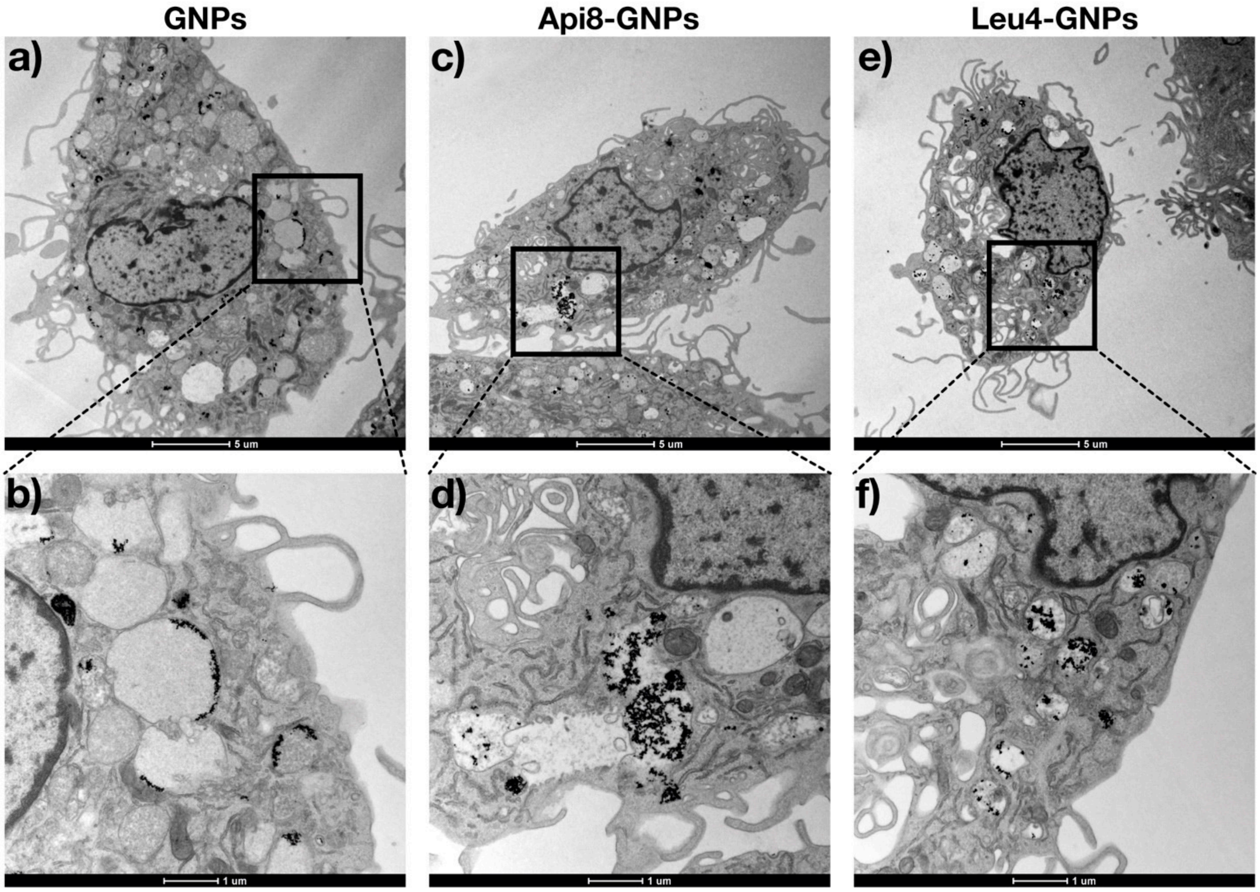

Figure S25. Representative TEM images of the interaction of different GNP formulations with human macrophages, derived from monocytes isolated from buffy coats. Scale bars: 5 μm (a,c,e); 1 μm (b,d,f).
